# Influence of Thermal Sensitivity of Functionally Graded Materials on Temperature during Braking

**DOI:** 10.3390/ma15030963

**Published:** 2022-01-26

**Authors:** Aleksander Yevtushenko, Katarzyna Topczewska, Przemysław Zamojski

**Affiliations:** Faculty of Mechanical Engineering, Bialystok University of Technology (BUT), 45C Wiejska Street, 15-351 Bialystok, Poland; a.yevtushenko@pb.edu.pl (A.Y.); p.zamojski@pb.edu.pl (P.Z.)

**Keywords:** thermal sensitivity, functionally graded materials, temperature, friction, braking

## Abstract

The model of the frictional heating process during single braking to determine the temperature of the functionally graded friction elements with an account of the thermal sensitivity of materials was proposed. The basis of this model is the exact solution of the one-dimensional thermal problem of friction during braking with constant deceleration. The formulas approximating the experimental data of the temperature dependencies of properties of the functionally graded materials (FGMs) were involved in the model to improve the accuracy of the achieved results. A comparative analysis was performed for data obtained for temperature-dependent FGMs and the corresponding data, calculated without consideration of thermal sensitivity. The results revealed that the assumption of thermal stability of FGMs during braking may cause a significant overestimation of temperature of the friction pair elements.

## 1. Introduction

During intensive braking, the volume temperature of the disc braking system may be higher than 450 °C [1] and the maximum temperature on the friction surfaces of the pad and the disc during single braking may even reach a level above 1000 °C [2]. In such severe conditions, the thermal and mechanical properties of materials may highly differ from the initial, reached at the ambient temperature. Therefore, in order to improve the theoretical analysis of the thermoelastic behavior of the braking systems, it is necessary to develop mathematical models taking into consideration the thermal sensitivity of friction materials. However, the introduction of the temperature-dependent properties in formulation of the thermal problems of friction leads to nonlinearity, so most of the published analyses have been performed using numerical methods, especially the finite element method [3,4]. One of the alternative techniques used to develop such nonlinear models of frictional heating is linearization by means of the Kirchhoff substitution [5]. This method relies on the reduction of the originally nonlinear heat conduction equation to the linear one. However, it works this way only for materials with simple nonlinearity, which means that their thermal conductivity and specific heat capacity are temperature-dependent, but the thermal diffusivity remains constant [6]. For materials with arbitrary nonlinearity, only the partial linearization by the Kirchhoff substitution of such a problem is possible; as a result, another nonlinear problem is obtained for which the method of solving is known [7]. The Kirchhoff transform has a similar effect in the heat conduction problems formulated for solids with simple nonlinear thermosensitivity under complex heat exchange. Some analytic–numerical methods for the solution of such problems have been proposed in the study [8]. Another technique to take into consideration the thermal sensitivity of materials is the method of successive approximations (iterations), in which the solution of the corresponding linear problem is adopted as the initial approximation, and then the solution found in the previous step is corrected. An iteration algorithm to solve the one-dimensional problem of heat conduction at braking has been proposed in the article [9]. Most models of the frictional heating process taking into account of the thermal sensitivity effect have been developed only for homogeneous materials. Modern friction materials often have a non-uniform, complex internal structure with a changing composition, microstructure, or porosity across the volume of material, such as functionally graded materials (FGMs), which are characterized by smooth variations of properties as a function of position along certain direction. In the case of devices operating at elevated temperatures, including braking systems, FGMs are primarily used in order to obtain high temperature resistance on the friction surface by dissipating heat from it to the inside of the element while maintaining low wear. FGMs of this type are usually two-component. Their friction surface is usually made of metal-ceramic, and the metal opposite surface (core) should have high thermal conductivity. The change of properties in the direction perpendicular to the friction surface is described by continuous functions, usually power or exponential. In the case of the latter, the material gradient parameters are responsible for the speed of transition from one material to another.

The problem of wear of an FGM strip with an account of the heating on sliding contact from friction has been considered in the study [10]. The exact solution of the problem was obtained with the help of the integral Laplace transform technique. It was assumed that the shear modulus is described by means of the function of the vertical coordinate. A comprehensive review of the literature concerning the thermal contact problems of frictional heating for functionally graded materials was provided in our previous article [11]. So far, investigations of the transient heat conduction in FGMs are limited, and most of them have ignored the temperature dependence of the material properties. Therefore, in general, those models are adequate only for relatively low temperatures in an FGM or the materials with insignificant thermal sensitivity. To accurately describe the thermomechanical behavior of FGMs, the temperature dependence of the material properties should be considered. The heat conduction problems formulated for FGMs with non-uniform spatially distributed and temperature-dependent properties are highly nonlinear. Nevertheless, several studies concerning such problems taking into consideration the thermal sensitivity of FGMs can be found, but most of them are solved by means of numerical or semi-analytical methods. The finite element method has been adopted in the paper [12], to perform the nonlinear transient thermal stress analysis of a thick-walled FGM cylinder with temperature-dependent material properties. Another nonlinear transient heat transfer and thermoelastic stress in thermosensitive functionally graded cylinder have been investigated using the Hermitian transfinite element method in the study [13]. The results showed that the effect of thermal sensitivity of materials has a significant influence on the thermal behavior of friction systems.

An analytical approach to solve the one-dimensional transient heat conduction problem for functionally graded materials with temperature-dependent properties has been presented in the article [14]. As for the analytical treatment, the temperature and thermal stress solutions have been obtained in approximate forms for a simplified, homogeneous, multi-layered model of materials. They concluded that the temperature dependence of the material properties is one of the most important factors in the accurate evaluation of temperature and stress distributions [14]. A similar multi-layered model was used to formulate another thermal problem of friction for a thermally sensitive FGM plate in the paper [15]. The authors made an attempt to optimize the functionally graded structure in order to enhance their thermal performance. The proper manufacturing process allows the design of an FGM according to the engineering demands by intentionally setting a specific distribution of the properties. A hybrid genetic algorithm has been developed for the optimization of the FGM composition with temperature-dependent material properties, in order to minimize the thermal stresses under steady-state thermal loads [15]. The optimum composition profile of the functionally graded materials for wide temperature ranges was also studied in the article [16]. The thermoelastic problem for functionally graded material with temperature-dependent properties was considered by means of the perturbation method. Additionally, the crack propagation path was predicted by introducing the fracture mechanics analysis. It was concluded that the proper selection of an FGM gradient can lead to a significant decrease in thermal stresses [16]. A transient thermoelastic behavior of the functionally graded plate with temperature-dependent properties due to a thermal shock was considered in the paper [17]. The temperature and thermal stress distributions in the Cu-W functionally graded composite were found by means of the semi-analytical micromechanical model.

The aim of this study was to investigate the influence of FGMs thermal sensitivity on the distribution of temperature in a disc brake system. This study is a continuation of our previous articles [11,18], which concern the transient thermal problem of friction under uniform sliding and during single braking with an exponential increase in the contact pressure. Due to the appearance of a high temperature level, there is a demand to improve the results by involving the variations of material properties dependent on the actual temperature, since the thermal sensitivity effect is particularly manifested in a high temperature range. In this article, the braking with constant deceleration is considered, when the nominal pressure is reached immediately at the beginning of the process, since the increase in the time of contact pressure growth causes a drop in the achieved temperature.

## 2. Statement to the Problem

To develop an analytical model of frictional heating process in the braking system, the following assumptions were taken into account:

1. The braking process with constant deceleration is considered;
2. At the initial time moment, the temperature of a brake is equal to the ambient temperature *T_a_*;
3. In the heat conduction equation, only the change in temperature gradient in the perpendicular direction to the disc-pad contact surfaces is taken into consideration;
4. The thermal contact on the friction surfaces is perfect, i.e., the temperatures of its contact surfaces are equal, and the sum of frictional heat fluxes intensities, acting along the normal direction to the contact surface to the insides of the elements equal to the specific friction power;
5. Due to the symmetry of the system with respect to the mid plane of the disc, when determining the brake temperature, the contact of one pad and a disc with half of its thickness is considered;
6. The pads and the disc are made of two-component thermally sensitive functionally graded materials, in such a way that their friction surfaces are materials with low thermal conductivity (i.e., cermet), while the core materials are characterized by higher thermal conductivity (titanium alloys, aluminum, etc.);
7. The thermal conductivity of the disc and pads materials increases exponentially with the distance from the contact surface;
8. The whole initial kinetic energy of the vehicle is transformed into heat during braking, neglecting the small part of energy associated with wear on the contact surfaces of the disc and pads;

Based on the assumptions (1)–(5), in order to determine the temperature of the disc-pad system, the scheme of sliding with linearly decreasing velocity of two semi-spaces z≥0 (disc) and z≤0 (pad) has been adopted. Initiated by the frictional heating temperature field of such a system at a given time instant t≥0 depends only on the distance from the friction surface in a perpendicular direction—independent variable z: T=T(z,t).

According to the assumption (6), the thermophysical properties of a friction pair are functions of temperature T:

(1) $$K_{l,m}=K_{l,m}(T),\;c_{l,m}=c_{l,m}(T),\;\rho_{l,m}=\rho_{l,m}(T),\;$$

where $K_{l,m}$, $c_{l,m}$ and $\rho_{l,m}$—thermal conductivity, specific heat capacity and density of the first (m=1) and second (m=2) component of the materials of the disc (l=1) and pad (l=2), respectively. Corresponding values at the initial system temperature $T=T_{0}$ are marked as follows:

(2) $$K_{l,m}^{\left(0\right)}\equiv K_{l,m}(T_{0}),c_{l,m}^{\left(0\right)}\equiv c_{l,m}(T_{0}),\rho_{l,m}^{\left(0\right)}\equiv \rho_{l,m}(T_{0}).$$

According to the mixture law, the effective specific heat capacities and densities were also determined:

(3) $$c_{l}^{\left(0\right)}=c_{l,2}^{\left(0\right)}V_{c}+(1-V_{c})c_{l,1}^{\left(0\right)},\;\rho_{l}^{\left(0\right)}=\rho_{l,2}^{\left(0\right)}V_{\rho }+(1-V_{\rho })\rho_{l,1}^{\left(0\right)},$$

where $V_{c}$, $V_{\rho }$—volume fractions of the phases $c_{l,m}^{\left(0\right)}$ and $\rho_{l,m}^{\left(0\right)}$, l=1,2, m=1,2, respectively.

Based on the assumption (7), the effective thermal conductivities $K_{l}$, l=1,2 of the disc and pad materials were established from the equations:

(4) $$K_{1}(z)=K_{1,1}e^{\gamma_{l}z},\;\;0\leq z\leq a,\;K_{2}(z)=K_{2,1}e^{-\gamma_{2}z},\;\;-a\leq z\leq 0,$$

where

(5) $$\gamma_{l}=\frac{\gamma_{l}^{*}}{a},\;\gamma_{l}^{*}=\ln\left(\frac{K_{l,2}^{\left(0\right)}}{K_{l,1}^{\left(0\right)}}\right),$$

(6) $$a=\max\{a_{1},\;a_{2}\},a_{l}=\sqrt{3k_{l}^{\left(0\right)}t_{s}},$$

(7) $$k_{l}^{\left(0\right)}=\frac{K_{l,1}^{\left(0\right)}}{c_{l}^{\left(0\right)}\rho_{l}^{\left(0\right)}},$$

and $t_{s}$—stop time, and parameters $a_{l}$, l=1,2 (6) are the thicknesses of the subsurface layers actively participating in heat absorption in the disc and pads, respectively (the so-called effective depth of heat transfer [19]). During braking with constant deceleration, the specific friction power decreases linearly from the nominal value $q_{0}$ to zero [20]:

(8) $$q(t)=q_{0}q^{*}(t),q_{0}=fp_{0}V_{0},q^{*}(t)=1-t\;t_{s}^{-1},\;0\leq t\leq t_{s},$$

(9) $$t_{s}=W_{0}\;Q_{0}^{-1},\;Q=q_{0}A_{a},A_{a}=0.5\beta (R_{e}^{2}-R_{i}^{2}),$$

where $A_{a}$—nominal area of the contact between the pad and the disc; f—friction coefficient; $p_{0}$—nominal pressure; $Q_{0}$—nominal friction power; 0≤β≤2π—nominal friction power; —cover angle of the pad; $R_{i}$ and $R_{e}$—respectively, the internal and external radii of the pads; $V_{0}$, $W_{0}$—the initial velocity and kinetic energy of the system, respectively. The latter, according to assumption (8), is equal to the total work of friction.

In order to solve the above-formulated nonlinear problem, we will use the idea of adapting an appropriate solution of the linear problem of thermal friction. This approach in the case of homogeneous materials was used in the studies [9,21].

## 3. Solution with Temperature-Independent FGMs Properties

The key element of the proposed approach is the precise solution of the linear thermal problem of friction during braking with constant deceleration. In the case of FGMs, such a solution for the above-adopted scheme of two sliding semi-spaces for the specific friction power q(t)(8) and (9) can be written in the form [18]:

(10) $$T(z,t)=T_{0}+\Theta (z,t),\;0\leq t\leq t_{s},$$

(11) $$\Theta (z,t)=\Lambda e^{-\gamma_{1}z/2}\left[\frac{e^{-\gamma_{1}z/2}}{\left(1+\gamma_{\varepsilon }K_{\varepsilon }\right)}q^{*}(t)+\frac{4}{\gamma_{\varepsilon }}\sum_{n=1}^{\infty }\frac{\varphi_{1}(z,\mu_{n})}{\Psi (\mu_{n})}G_{n}(t)\right],\;z\geq 0,\;$$

(12) $$\Theta (z,t)=\Lambda e^{\gamma_{1}z/2}\left[\frac{e^{\gamma_{2}z/2}}{\left(1+\gamma_{\varepsilon }K_{\varepsilon }\right)}q^{*}(t)+\frac{4}{\gamma_{\varepsilon }}\sum_{n=1}^{\infty }\frac{\varphi_{2}(z,\mu_{n})}{\Psi (\mu_{n})}G_{n}(t)\right],\;z\leq 0,$$

(13) $$G_{n}(t)=e^{-p_{n}t}-\frac{\left(1-e^{-p_{n}t}\right)}{p_{n}t_{s}},p_{n}=\frac{1}{4}k_{1}{\left(\gamma_{1}\mu_{n}\right)}^{2},$$

(14) $$\varphi_{1}(z,\mu_{n})=J_{1}(\gamma_{\varepsilon }\mu_{n})J_{1}(\mu_{n}e^{-\gamma_{1}z/2}),\;\varphi_{2}(z,\mu_{n})=J_{1}(\mu_{n})J_{1}(\gamma_{\varepsilon }\mu_{n}e^{\gamma_{2}z/2}),$$

(15) $$\Psi (\mu_{n})=\mu_{n}^{2}[(1+\gamma_{\varepsilon }K_{\varepsilon })J_{0}(\mu_{n})J_{0}(\gamma_{\varepsilon }\mu_{n})-(\gamma_{\varepsilon }+K_{\varepsilon })J_{1}(\mu_{n})J_{1}(\gamma_{\varepsilon }\mu_{n})],$$

(16) $$K_{\varepsilon }=K^{*}{\left(k^{*}\right)}^{-1/2},\;\gamma_{\varepsilon }=\gamma^{*}{\left(k^{*}\right)}^{1/2},$$

(17) $$\Lambda =\frac{q_{0}}{\gamma_{2}K_{2,1}^{\left(0\right)}},\;K^{*}=\frac{K_{1,1}^{\left(0\right)}}{K_{2,1}^{\left(0\right)}},\;k^{*}=\frac{k_{1}^{\left(0\right)}}{k_{2}^{\left(0\right)}},\;\gamma^{*}=\frac{\gamma_{1}}{\gamma_{2}},$$

where $\mu_{n}>0,\;n=1,2,3,\ldots$, are the real roots of the functional equation:

(18) $$J_{0}(\gamma_{\varepsilon }\mu_{n})J_{1}(\mu_{n})+K_{\varepsilon }J_{0}(\mu_{n})J_{1}(\gamma_{\varepsilon }\mu_{n})=0.$$

$J_{k}(x)$—are the Bessel functions of the first kind of the *k*th order [22].

The temperature of the friction surfaces of both elements, in accordance with the assumption (4) of their perfect thermal contact of friction, should be the same. Substituting z=0 in Equations (10)–(12) and (14), the following were obtained:

(19) $$T(t)\equiv T(0^{\pm },t)=T_{0}+\Theta (t),\;0\leq t\leq t_{s},$$

(20) $$\Theta (t)\equiv \Theta (0^{\pm },t)=\Lambda \left[\frac{q^{*}(t)}{\left(1+\gamma_{\varepsilon }K_{\varepsilon }\right)}+\frac{4}{\gamma_{\varepsilon }}\sum_{n=1}^{\infty }\frac{\hat{\varphi }(\mu_{n})}{\Psi (\mu_{n})}G_{n}(t)\right],\;0\leq t\leq t_{s},$$

where

(21) $$\hat{\varphi }(\mu_{n})=J_{1}(\gamma_{\varepsilon }\mu_{n})J_{1}(\mu_{n}).$$

Dimensionless variables and parameters were introduced:

(22) $$\zeta =\frac{z}{a},\;\tau =\frac{k_{1}t}{a^{2}},\;\tau_{s}=\frac{k_{1}t_{s}}{a^{2}},\;\Theta_{0}=\frac{q_{0}a}{K_{1,1}^{\left(0\right)}},\;\Theta^{*}=\frac{\Theta }{\Theta_{0}},$$

where parameters a and $q_{0}$ were determined accordingly from Formulas (6) and (8). Taking into account the indications (22) in Formulas (11)–(14), the dimensionless temperature rise of the friction pair elements can be presented in the form:

(23) $$\Theta^{*}(\zeta ,\tau )=\frac{K_{0}^{*}}{\gamma_{2}^{*}}e^{-\gamma_{1}^{*}\zeta /2}\left[\frac{e^{-\gamma_{1}^{*}\zeta /2}}{\left(1+\gamma_{\varepsilon }K_{\varepsilon }\right)}q^{*}(\tau )+\frac{4}{\gamma_{\varepsilon }}\sum_{n=1}^{\infty }\frac{\varphi_{1}^{*}(\zeta ,\mu_{n})}{\Psi (\mu_{n})}G_{n}(\tau )\right],\zeta \geq 0,\;0\leq \tau \leq \tau_{s},$$

(24) $$\Theta^{*}(\zeta ,\tau )=\frac{K_{0}^{*}}{\gamma_{2}^{*}}e^{\gamma_{2}^{*}\zeta /2}\left[\frac{e^{\gamma_{2}^{*}\zeta /2}}{\left(1+\gamma_{\varepsilon }K_{\varepsilon }\right)}q^{*}(\tau )+\frac{4}{\gamma_{\varepsilon }}\sum_{n=1}^{\infty }\frac{\varphi_{2}^{*}(\zeta ,\mu_{n})}{\Psi (\mu_{n})}G_{n}(\tau )\right],\;\zeta \geq 0,\;0\leq \tau \leq \tau_{s},$$

where:

(25) $$\varphi_{1}^{*}(\zeta ,\mu_{n})=J_{1}(\gamma_{\varepsilon }\mu_{n})J_{1}(\mu_{n}e^{-\gamma_{1}^{*}\zeta /2}),\;\varphi_{2}(\zeta ,\mu_{n})=J_{1}(\mu_{n})J_{1}(\gamma_{\varepsilon }\mu_{n}e^{\gamma_{2}^{*}\zeta /2}),$$

(26) $$G_{n}(\tau )=e^{-\lambda_{n}\tau }-\frac{1}{\lambda_{n}\tau_{s}}(1-e^{-\lambda_{n}\tau }),\;\lambda_{n}=\frac{1}{4}{\left(\gamma_{1}^{*}\mu_{n}\right)}^{2},$$

and the remaining functions as well as parameters are given by Formulas (15)–(18). Substituting ζ=0 in Formulas (23)–(25), the dimensionless rise of the temperature on the friction surfaces was obtained:

(27) $$\Theta^{*}(\tau )\equiv \Theta^{*}(0^{\pm },\tau )=\frac{K_{0}^{*}}{\gamma_{2}^{*}}\left[\frac{q^{*}(\tau )}{\left(1+\gamma_{\varepsilon }K_{\varepsilon }\right)}+\frac{4}{\gamma_{\varepsilon }}\sum_{n=1}^{\infty }\frac{\hat{\varphi }(\mu_{n})}{\Psi (\mu_{n})}G_{n}(\tau )\right],\;0\leq \tau \leq \tau_{s},$$

Based on Fourier’s law, the intensity of heat fluxes directed along the normal to the contact surface z=0 towards the insides of the friction pair elements were defined:

(28) $$q_{l}(t)={{\left(-1\right)}^{l}K_{l,1}^{\left(0\right)}\frac{\partial T(z,t)}{\partial z}|}_{z=0^{\pm }},\;0\leq t\leq t_{s},\;l=1,2.$$

Taking into account the indications (22) dimensionless intensities of heat fluxes $q_{l}^{*}=q_{l}q_{0}^{-1}$, l=1,2 were written as:

(29) $$q_{1}^{*}(\tau )={-\frac{\partial \Theta^{*}(\zeta ,\tau )}{\partial \zeta }|}_{\zeta =0^{+}},\;q_{2}^{*}(\tau )={\frac{\partial \Theta^{*}(\zeta ,\tau )}{K_{0}^{*}\partial \zeta }|}_{\zeta =0^{-}},\;0\leq \tau \leq \tau_{s}.$$

After differentiating the solution (23)–(26) with respect to the variable ζ and subsequent substitution of the found derivatives to the right side of Formula (30), the following was found:

(30) $$q_{1}^{*}(\tau )=\frac{\gamma_{\varepsilon }K_{\varepsilon }}{\left(1+\gamma_{\varepsilon }K_{\varepsilon }\right)}q^{*}(\tau )+2\gamma_{\varepsilon }K_{\varepsilon }\sum_{n=1}^{\infty }\frac{\tilde{\varphi }(\mu_{n})}{\tilde{\Psi }(\mu_{n})}G_{n}(\tau ),\;0\leq \tau \leq \tau_{s},$$

(31) $$q_{2}^{*}(\tau )=\frac{1}{\left(1+\gamma_{\varepsilon }K_{\varepsilon }\right)}q^{*}(\tau )+2\sum_{n=1}^{\infty }\frac{\tilde{\varphi }(\mu_{n})}{\tilde{\Psi }(\mu_{n})}G_{n}(\tau ),\;0\leq \tau \leq \tau_{s},$$

where:

(32) $$\tilde{\varphi }(\mu_{n})=J_{0}(\gamma_{\varepsilon }\mu_{n})J_{1}(\mu_{n}),\;\tilde{\Psi }(\mu_{n})=\mu_{n}^{-1}\;\Psi (\mu_{n}),$$

and functions $\Psi (\mu_{n})$ and $G_{n}(\tau )$ can be determined from Equations (15) and (26), respectively.

It should be noted that in the case of homogeneous materials ($\gamma_{i}\to 0,\;i=1,2$) of the disc and pads, the dimensionless temperature rise during braking with a constant deceleration has the form [23]:

(33) $$\Theta^{*}(\zeta ,\tau )=\frac{2K^{*}\sqrt{\tau }}{\left(1+K_{\varepsilon }\right)}\left\{\mathrm{ierfc}\left(\frac{\zeta }{2\sqrt{\tau }}\right)-\frac{\tau }{\tau_{s}}\left[\left(1+\frac{\zeta^{2}}{6\tau }\right)\mathrm{ierfc}\left(\frac{\zeta }{2\sqrt{\tau }}\right)-\frac{e^{-\frac{\zeta^{2}}{4\tau }}}{3\sqrt{\pi }}\right]\right\},\;\zeta \geq 0,\;0\leq \tau \leq \tau_{s},$$

(34) $$\Theta^{*}(\zeta ,\tau )=\frac{2K^{*}\sqrt{\tau }}{\left(1+K_{\varepsilon }\right)}\left\{\mathrm{ierfc}\left(-\frac{\zeta }{2}\sqrt{\frac{k_{0}^{*}}{\tau }}\right)-\frac{\tau }{\tau_{s}}\left[\left(1+\frac{\zeta^{2}k_{0}^{*}}{6\tau }\right)\mathrm{ierfc}\left(-\frac{\zeta }{2}\sqrt{\frac{k_{0}^{*}}{\tau }}\right)-\frac{e^{-\frac{\zeta^{2}k_{0}^{*}}{4\tau }}}{3\sqrt{\pi }}\right]\right\},\;\zeta \leq 0,\;0\leq \tau \leq \tau_{s},$$

where $\mathrm{ierfc}(x)=\pi^{-1/2}e^{-x^{2}}-x\;\mathrm{erfc}(x)$, erfc(x)=1−erf(x), erf(x)—Gaussian error function. For ζ=0 from Equations (33) and (34), the known solution of Fazekas was obtained [24]:

(35) $$\Theta^{*}(\tau )=\frac{2K^{*}}{\left(1+K_{\varepsilon }\right)}\sqrt{\frac{\tau }{\pi }}\left(1-\frac{2\tau }{3\tau_{s}}\right),\;0\leq \tau \leq \tau_{s}.$$

## 4. Volume Temperature

With the given input parameters, solutions (19)–(27) make it possible to find the space–time distribution of the temperature inside and its evolution on the friction surfaces of the pad and disc, made of thermally insensitive FGMs. In order to take into account the thermal sensitivity of materials determining the temperature of the braking system using the above-mentioned solutions, the thermal conductivities $K_{l,m}^{\left(0\right)}$, specific heat capacities $c_{l,m}^{\left(0\right)}$ and densities $\rho_{l,m}^{\left(0\right)}$ at the initial temperature $T_{0}$ were replaced with corresponding values $K_{l,m}^{\left(\vartheta_{l}\right)}$, $c_{l,m}^{\left(\vartheta_{l}\right)}$ and $\rho_{l,m}^{\left(\vartheta_{l}\right)}$ found from Equations (1) and (2) for the volume temperature of the pad and the disc during braking [2,9]:

(36) $$\vartheta_{l}=T_{0}+{\hat{\vartheta }}_{l},\;l=1,2,$$

where:

(37) $${\hat{\vartheta }}_{l}=\frac{2\alpha_{l}\;W_{0}}{3G_{l}c_{l}^{\left(0\right)}},$$

(38) $$G_{l}=A_{a}a_{l}\rho_{l}^{\left(0\right)},$$

$a_{l}$—the effective depths of heat penetration (6), $\alpha_{1}=\alpha$, $\alpha_{2}=1-\alpha$, 0≤α≤1—heat partition ratio. Based on Formulas (30)–(32), the heat partition ratio was calculated from the formula:

(39) $$\alpha \equiv \frac{q_{1}(t)}{q(t)}\approx \frac{\gamma_{\varepsilon }K_{\varepsilon }}{1+\gamma_{\varepsilon }K_{\varepsilon }}.$$

## 5. Numerical Analysis

The calculations were performed for the friction pair, one element of which was made of aluminium oxide Al_2_O_3_ (friction surface) and cooper Cu (core) [25]. The friction surface and core of the second element are manufactured of zirconium dioxide ZrO_2_ and titanium alloy Ti-6Al-4V [14]. The temperature-dependent properties of these materials are as follows:

Al_2_O_3_ [26,27,28]

(40) $$K_{1,1}(T)=39.717-0.130T+4.463\cdot 10^{-4}T^{2}-2.836\cdot 10^{-7}T^{3}+1.941\cdot 10^{-10}T^{4},$$

(41) $$c_{1,1}(T)=680.72+2.432T-0.53\cdot 10^{-2}T^{2}+0.6\cdot 10^{-5}T^{3}-0.4\cdot 10^{-8}T^{4}+10^{-12}T^{5},$$

(42) $$\rho_{1,1}(T)=3992.2-0.062T-0.6\cdot 10^{-4}T^{2}+0.4\cdot 10^{-7}T^{3}-0.9\cdot 10^{-11}T^{4},$$

Cu [17,29]

(43) $$K_{1,2}(T)=31.985+0.0099\;T-0.1\cdot 10^{-5}T^{2},$$

(44) $$c_{1,2}(T)=523.3+1.4726\;T-0.0024T^{2}+0.2\cdot 10^{-5}T^{3}-0.5\cdot 10^{-9}T^{4},$$

(45) $$\rho_{1,2}(T)=492.45-0.01\;T-0.1\cdot 10^{-5}T^{2},$$

ZrO_2_ [27,30,31]

(46) $$K_{2,1}(T)=1.9365+0.7\cdot 10^{-4}T+0.5\cdot 10^{-6}\;T^{2}-0.2\cdot 10^{-9}T^{3},$$

(47) $$c_{2,1}(T)=437.96+0.7767T-0.17\cdot 10^{-2}T^{2},$$

(48) $$\rho_{2,1}(T)=6104.6-0.1212T-0.4\cdot 10^{-4}T^{2}+0.3\cdot 10^{-7}T^{3}-0.1\cdot 10^{-10}T^{4},$$

Ti-6Al-4V [32,33]

(49) $$K_{2,2}(T)=6.6926+8.9177\cdot 10^{-3}\;T+6.8432\cdot 10^{-6}T^{2},$$

(50) $$c_{2,2}(T)=529.9316+0.4154T-4.01646\cdot 10^{-4}T^{2}+1.6364\cdot 10^{-7}T^{3},$$

(51) $$\rho_{2,2}(T)=4434-0.1088T-0.8\cdot 10^{-4}T^{2}+10^{-7}T^{3}-0.6\cdot 10^{-10}T^{4}.$$

**Figure 1:**
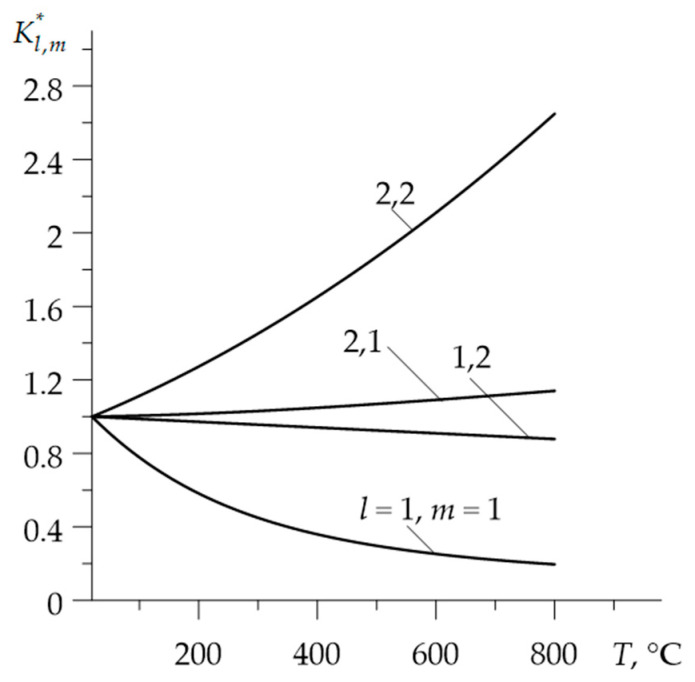
Dependencies of the dimensionless thermal conductivities $K_{l,m}^{*}$ on temperature T.

**Figure 2:**
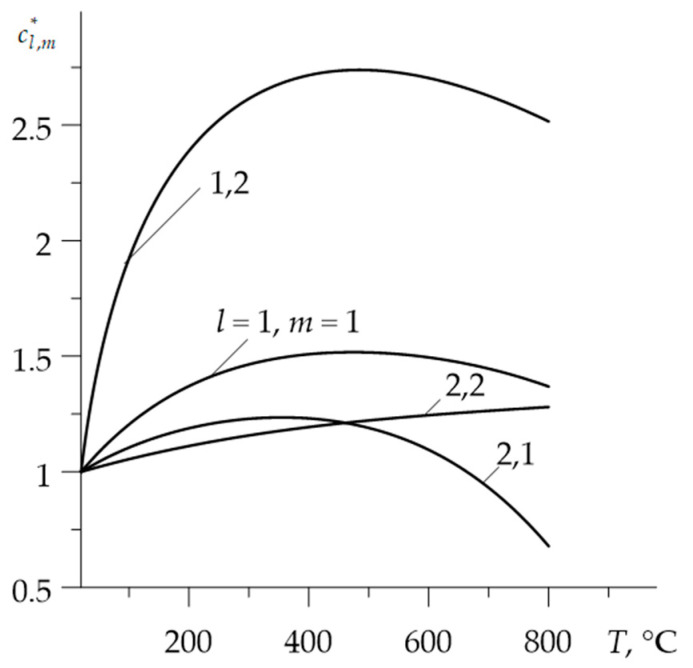
Dependencies of the dimensionless specific heat capacities $c_{l,m}^{*}$ on temperature T.

**Figure 3:**
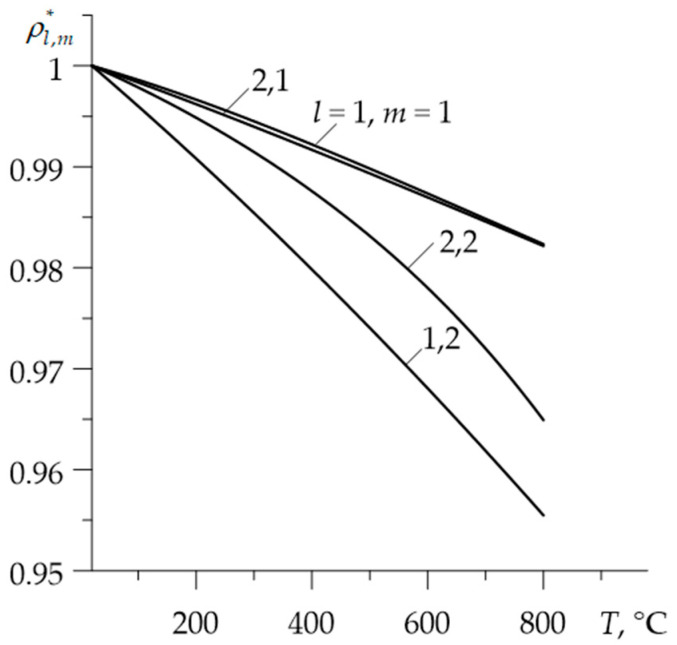
Dependencies of the dimensionless densities $\rho_{l,m}^{*}$ on temperature T.

Graphs of dimensionless functions $K_{l,m}^{*}=K_{l,m}(T)/K_{l,m}^{\left(0\right)}$, $c_{l,m}^{*}=c_{l,m}(T)/c_{l,m}^{\left(0\right)}\;$ and $\rho_{l,m}^{*}=\rho_{l,m}(T)/\rho_{l,m}^{\left(0\right)}$ are illustrated in the Figure 1, Figure 2 and Figure 3.

The calculations were performed according to the following scheme:

(1) (1) the values of the input parameters were given (Table 1), and then from Equations (8) and (9) the area of the nominal contact was calculated $A_{a}=0.0022\;m^{2}$, specific friction power $q_{0}=3.87\;\mathrm{MW}\;m^{-2}$, friction power $Q_{0}=8510\;\;W$ and stop time $t_{s}=12.1\;s$;
(2) using the dependencies (40)–(51) the materials properties $K_{l,m}^{\left(0\right)}$, $c_{l,m}^{\left(0\right)}$ and $\rho_{l,m}^{\left(0\right)}$, l, m=1,2 at the initial temperature $T_{0}=20{\;}^{\circ }C$ were established (Table 2);
(3) the effective values of: the specific heat $c_{l}^{\left(0\right)}$, density $\rho_{l}^{\left(0\right)}$, thermal diffusivity $k_{l}^{\left(0\right)}$, the effective depths of heat penetration $a_{l}$ and the dimensionless gradient parameters of materials $\gamma_{l}^{*}$, l=1,2 were found from Equations (3) and (5)–(7). Then, the dimensionless parameters $K_{\varepsilon }$ and $\gamma_{\varepsilon }$ were determined from the Formulas (16) and (17), and also the weight $G_{l}$ and heat partition ratios $\alpha_{l}$, l=1,2 were calculated from the Equations (38) and (39) (Table 3);
(4) the volume temperature values $\vartheta_{1}^{\left(0\right)}=471.97$ of the disc and $\vartheta_{2}^{\left(0\right)}=260.92$ the pad were obtained from the Equations (36) and (37);
(5) the values of materials properties $K_{l,m}^{\left(\vartheta_{l}^{\left(0\right)}\right)}$, $c_{l,m}^{\left(\vartheta_{l}^{\left(0\right)}\right)}$, $\rho_{l,m}^{\left(\vartheta_{l}^{\left(0\right)}\right)}$, l,m=1,2, corresponding to the volume temperature $\vartheta_{l}^{\left(0\right)}$ were determined from the Formulas (40)–(51);
(6) the steps (3)–(5) were repeated resulting in the corrected values for the volume temperature $\vartheta_{l}^{\left(1\right)}=624.93$, and $\vartheta_{2}^{\left(1\right)}=292.98$;
(7) by means of the formula $\vartheta_{l}=0.5(\vartheta_{l}^{\left(0\right)}+\vartheta_{l}^{\left(1\right)})$, l=1,2 final values of the volume temperature $\vartheta_{1}=548.45{\;}^{\circ }C,$ and $\vartheta_{2}=267.95{\;}^{\circ }C$ were found;
(8) based on the dependencies (40)–(51) the values of materials properties $K_{l,m}^{\left(\vartheta_{l}\right)}$, $c_{l,m}^{\left(\vartheta_{l}\right)}$,$\rho_{l,m}^{\left(\vartheta_{l}\right)}$, l,m=1,2 corresponding to the volume temperature $\vartheta_{l}$ were established (Table 4) and other parameters necessary to perform the calculations (Table 5);
(9) the temperature field $\Theta^{*}(\zeta ,\tau )$ (23)–(26), the temperature evolution $\Theta^{*}(\tau )$ (27), and temporal profiles of heat fluxes intensities $q_{l}^{*}(\tau )$, l=1,2 (30)–(32) were determined.

In order to calculate the values of Bessel functions $J_{k}(x)$, k=0, 1 the programs BESSJ0 and BESSJ1 from the Numerical Recipes package [34] were used. The roots of the characteristic Equation (18) were searched for by the bisection method with the RTBIS program from this package. In summation of the series in solutions (23), (24), and (30), (31) was performed with an accuracy of $5\times 10^{-5}$. For this accuracy, the minimum number of components was equal to 70.

**Figure 4:**
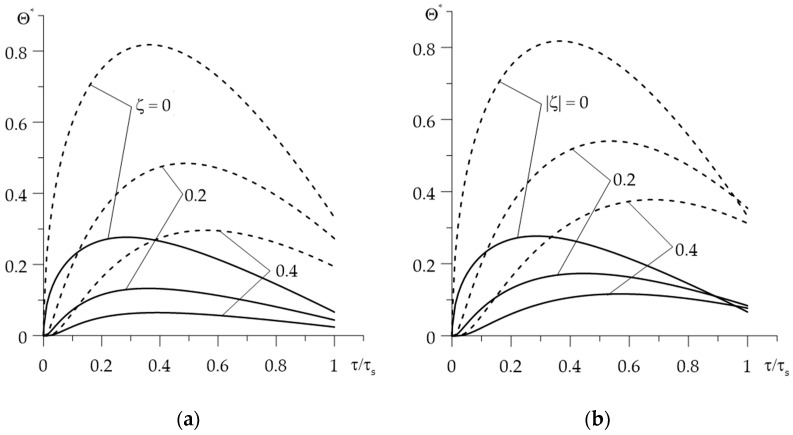
Evolutions of the dimensionless temperature $\Theta^{*}(\zeta ,\tau )$ during braking at different distances ζ from the surface of friction with (solid lines) and without (dashed lines) taking into account the thermal sensitivity of the materials: (**a**) Al_2_O_3_—Cu; (**b**) ZrO_2_—Ti-6Al-4V.

Changes in the dimensionless temperature rise $\Theta^{*}(\zeta ,\tau )$ during braking, at few selected distances from the contact surface are presented in Figure 4. The temperature calculated with an account of the thermal sensitivity of the materials (solid lines) is significantly lower in both friction elements compared to the results achieved without taking into account the temperature dependencies of FGMs properties (dashed lines). The maximum dimensionless temperature on the contact surface ζ=0 without and taking into account the thermal sensitivity of the materials are 0.816 and 0.277, respectively (reduction of about 2.94 times) and are reached at the time moments $\tau_{\max}=0.37$ and $\tau_{\max}=0.29$ (reduction of 21.6%).

**Figure 5:**
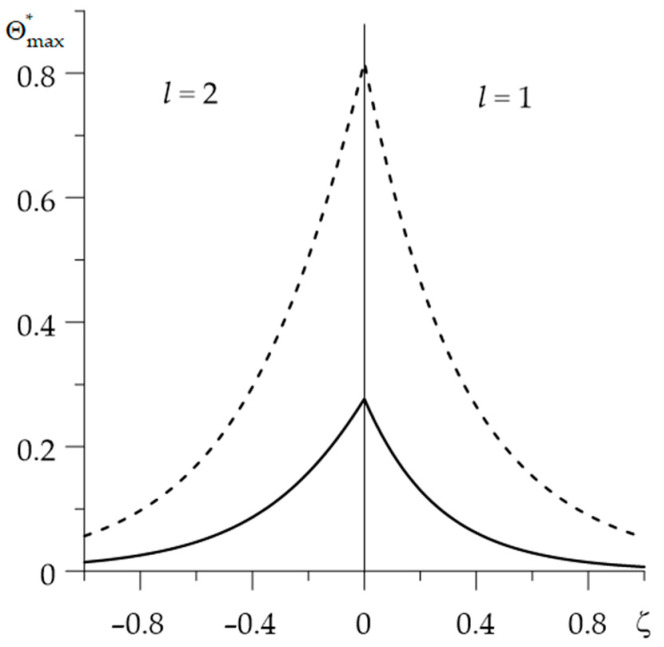
Distribution of the dimensionless temperature $\Theta_{\max}^{*}(\zeta )=\Theta^{*}(\zeta ,\tau_{\max})$ reached at the time moment $\tau =\tau_{\max}$ along the distance ζ from the surface of friction with (solid lines) and without (dashed lines) taking into account the thermal sensitivity of the materials.

Increasing the distance from the contact surface ζ=0, the temperature level of both elements drops (Figure 5). The temperature of components made of thermally sensitive materials is lower than their temperature, found for the constant material properties. The greatest difference between these results is on the contact surface.

**Figure 6:**
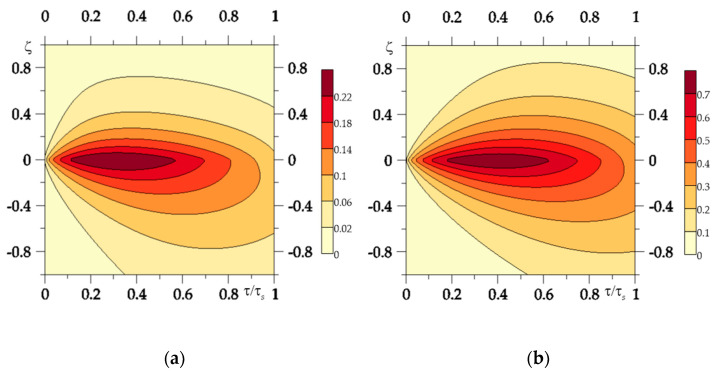
Isotherms of the dimensionless temperature $\Theta^{*}(\zeta ,\tau )$ for: (**a**) thermally sensitivity materials; (**b**) materials with properties at the initial temperature.

The conclusions established on the basis of Figure 4 and Figure 5 confirm the results of the calculations, presented in Figure 6. It shows the dimensionless temperature isotherms $\Theta^{*}(\zeta ,\tau )$. It can be seen that the effective depth of heat transfer is much greater in the case that material properties remain unchanged under the influence of temperature, than in the case of considering the thermally sensitive FGMs. This effect is most noticeable for the first one (l=1), the Al_2_O_3_-Cu element. This result is also confirmed by the parameter values $a_{l}$, l=1,2 presented in Table 3 and Table 5.

**Figure 7:**
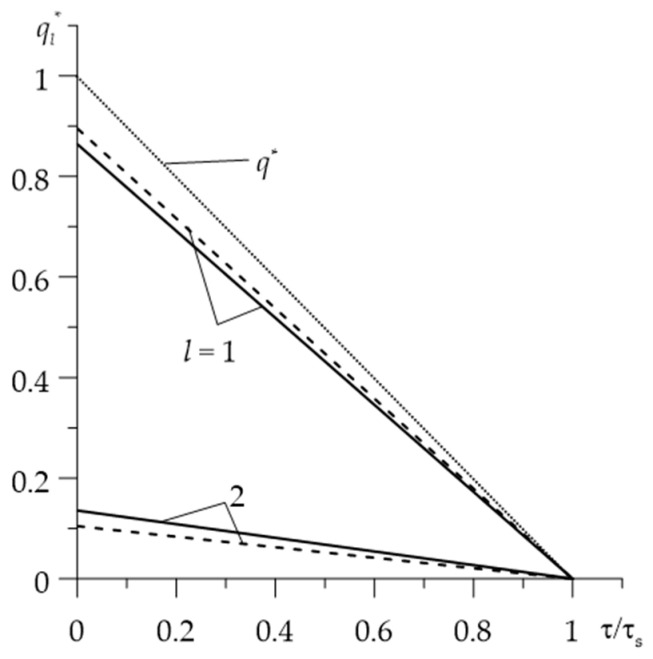
Temporal profiles of the dimensionless heat fluxes $q_{l}^{*}(\tau )$, l=1,2 during braking with (continuous lines) and without (dashed lines) taking into account the thermal sensitivity of the materials. Dotted lines represent the dimensionless specific power of friction $q^{*}$.

The time profiles of the dimensionless intensities of heat fluxes $q_{l}^{*}(\tau )$, l=1,2 are shown in Figure 7. They decrease linearly during the braking process from the maximum value at the initial moment to zero at the stop. Most of the frictional heat generated is absorbed by the first element (l=1) Al_2_O_3_-Cu. The linear change in $q_{l}^{*}(\tau )$ is the result of the specific friction power $q^{*}(\tau )$ (8), which decreases linearly during braking with a constant deceleration, and the requirement to meet the boundary condition $q_{1}^{*}(\tau )+q_{2}^{*}(\tau )=q^{*}(\tau )$, $0\leq \tau \leq \tau_{s}$. The influence of thermal sensitivity on the intensity of heat fluxes is much smaller than on the temperature. For thermally sensitive materials, the maximum values of the intensity of heat fluxes are $q_{1,\max}^{*}=0.864$ and $q_{2,\max}^{*}=0.136$, and for constant properties of the materials, we have $q_{1,\max}^{*}=0.895$ and $q_{2,\max}^{*}=0.105$.

## 6. Conclusions

A calculation scheme was proposed to determine the temperature field of the friction elements of a disc brake, taking into account the changes in the FGMs properties depending on the actual temperature. The main part of the scheme was the adaptation of a linear solution (with temperature-independent material properties) to the thermal problem of friction during braking to thermally sensitive FGMs. A numerical analysis was performed in the case of braking with constant deceleration of elements made of two-component functionally graded materials with exponential variations in thermal conductivities in the axial direction, across the volume of the materials. It was found that:

- the influence of thermal sensitivity on the temperature of FGMs may be more significant than in the case of homogeneous materials;
- for the selected friction pair, taking into account the thermal sensitivity caused an almost threefold reduction in the maximum temperature in comparison to the appropriate temperature values, found with the same properties of the materials;
- the influence of thermal sensitivity on the intensity of heat fluxes directed from the friction surface to the interior of the friction pair elements is insignificant. This means that to estimate the amount of heat absorbed by the individual elements of the friction pair, appropriate solutions to linear problems can be used.

A verification of the developed theoretical model based on empirical results would be advisable. However, no information on this kind of experimental data has been found in the literature. In particular, it concerns the frictional heating of braking systems with friction elements made of thermally sensitive FGMs. Therefore, the verification of the exact solution was obtained carried out by determining from it, in cases of limit parameters, known solutions of other authors for homogeneous materials, which were verified with appropriate experimental data. A new element, significantly differentiating the results of a given article from those published earlier by us, is the incorporation in the model of the possibility of changing the frictional properties of FGMs under temperature influence. This model includes many new elements, such as determining the intensity of heat fluxes to obtain the form of the heat partition ratio, finding the volume temperature of FGMs, developing a calculation algorithm that takes into account the thermal sensitivity of all materials components, etc. We have shown that taking into consideration the thermal sensitivity of materials can significantly reduce the surface temperature contact of the pad and disc. We proposed a theoretical computational model. We hope that it will be verified with the data obtained from other authors’ research positions. An indirect confirmation of the correctness of our model is also the time profiles of temperature and heat fluxes obtained on its basis, characteristic for braking with a constant deceleration.

It should be noted that all three of our papers constitute a monothematic cycle of interrelated research. We also want to develop a suitable model for braking systems operating in a short-term, repetitive mode. The problem of lowering the temperature level in such systems is up to date.

## Figures and Tables

**Table 1:** Input parameters.

Friction Coefficient f	Nominal Pressure $p_{0},\;\mathrm{MPa}$	Initial Sliding Speed $V_{0}^{},\;{\mathrm{ms}}^{-1}$	Initial Kinetic Energy $W_{0}^{},\;\mathrm{kJ}$	Outer Radius $R_{e}^{},\;\mathrm{mm}$	Inner Radius $R_{i}^{},\;\mathrm{mm}$	Initial Temperature $T_{0}^{},\;{}^{{}^{\circ}}C$
0.27	0.602	23.8	103.54	37.5	26.5	20

**Table 2:** Material properties at the initial temperature $T_{0}$.

Element Index	Material Index	Material	Thermal Conductivity $K_{l,m}^{\left(0\right)},\;\;Wm^{-1}K^{-1}$	Specific Heat Capacity $c_{l,m}^{\left(0\right)},\;J\;kg^{-1}K^{-1}$	Density $\rho_{l,m}^{\left(0\right)},\;\;\mathrm{kg}m^{-3}$
*l* = 1	*m* = 1	Al_2_O_3_	37.24	727.29	3990.92
	*m* = 2	Cu	402.65	147.35	8947.92
*l* = 2	*m* = 1	ZrO_2_	1.94	452.83	6102.16
	*m* = 2	Ti-6Al-4V	6.87	538.08	4431.79

**Table 3:** Calculated parameters at the initial temperature $T_{0}$.

Element Index	*l* = 1	*l* = 2
$c_{l}^{\left(0\right)},\;J\;{\mathrm{kg}}^{-1}K^{-1}$	437.3	495.5
$\rho_{l}^{\left(0\right)},\;{\mathrm{kgm}}^{-3}$	6469.4	5267
$k_{l}^{\left(0\right)}\times \;10^{6},\;m^{2}\;s^{-1}$	13.2	0.743
$\gamma_{l}^{*}$	2.381	1.266
$a_{l}^{},\;\mathrm{mm}$	21.854	5.193
$G_{l}^{},\;\mathrm{kg}$	0.3127	0.0605
$\alpha_{l}^{}$	0.896	0.104

**Table 4:** Material properties at volume temperature $\vartheta_{l}$, l=1,2.

Element Index	Material Index	Material	Thermal Conductivity $K_{l,m}^{\left(\vartheta_{l}\right)},\;\;{\mathrm{Wm}}^{-1}K^{-1}$	Specific Heat Capacity $c_{l,m}^{\left(\vartheta_{l}\right)},\;J\;{\mathrm{kg}}^{-1}K^{-1}$	Density $\rho_{l,m}^{\left(\vartheta_{l}\right)},\;\;{\mathrm{kgm}}^{-3}$
*l* = 1	*m* = 1	Al_2_O_3_	10.19	1097.93	3945.59
	*m* = 2	Cu	367.15	401.89	8690.20
*l* = 2	*m* = 1	ZrO_2_	1.99	552.67	6069.84
	*m* = 2	Ti-6Al-4V	9.57	615.44	4399.06

**Table 5:** Calculated parameters at volume temperature $\vartheta_{l}$, l=1,2.

Element Index	*l* = 1	*l* = 2
$c_{l}^{\left(\vartheta_{l}\right)},\;J\;{\mathrm{kg}}^{-1}K^{-1}$	749.7	584.9
$\rho_{l}^{\left(\vartheta_{l}\right)},\;\;{\mathrm{kgm}}^{-3}$	6317.2	5233.8
$k_{l}^{\left(\vartheta_{l}\right)}\times \;10^{6},\;m^{2}\;s^{-1}$	2.15	0.65
$\gamma_{l}^{*}$	3.585	1.583
$a_{l}^{},\;\mathrm{mm}$	8.834	4.854
$G_{l}^{},\;\mathrm{kg}$	0.1234	0.0562
$\alpha_{l}^{}$	0.863	0.137

## Data Availability

No new data were created or analyzed in this study. Data sharing is not applicable to this article.

## Nomenclature

$a_{l}$	Effective depth of heat penetration (m)
$A_{a}$	Area of the nominal contact region ($m^{2}$)
$c_{l,m}$	Specific heat capacity ($J\;{\mathrm{kg}}^{-1}K^{-1}$)
f	Coefficient of friction (dimensionless)
$G_{l}$	Weight of the friction elements (kg)
$J_{k}(\cdot )$	The Bessel functions of the first kind of the *k*th order
$k_{l,m}$	Thermal diffusivity ($m^{2}s^{-1}$)
$K_{l,m}$	Thermal conductivity ($W\;m^{-1}K^{-1}$)
p	Contact pressure (Pa)
$p_{0}$	Nominal value of the contact pressure (Pa)
$R_{e}$	External radius of the pads (m)
$R_{i}$	Internal radius of the pads (m)
q	Specific power of friction ($W\;m^{-2}$)
*q*_0_	Nominal value of the specific power of friction ($W\;m^{-2}$)
$Q_{0}$	Nominal friction power (W)
t	Time (s)
$t_{s}$	Stop time (s)
T	Temperature (${}^{\circ }C$)
$T_{0}$	Initial temperature (${}^{\circ }C$)
V	Velocity ($m\;s^{-1}$)
$V_{c},\;V_{\rho }$	Volume fractions of the material phases
$V_{0}$	Initial velocity ($m\;s^{-1}$)
$W_{0}$	Initial kinetic energy of the system (J)
z	Spatial coordinate in axial direction (m)
lower l	Number of the main (l=1) and frictional (l=2) elements of the friction pair
lower m	Number of the component material m=1, 2 of selected friction element
$\alpha_{l}^{}$	Heat partition ratio (dimensionless)
β	Cover angle of the pads (rad)
$\gamma_{l}^{}$	Parameter of material gradient ($m^{-1}$)
$\gamma_{l}^{*}$	Parameter of material gradient (dimensionless)
$\Theta_{l}$	Temperature rise (${}^{\circ }C$)
$\Theta_{l}^{*}$	Temperature rise (dimensionless)
$\Theta_{0}$	Temperature scaling factor (${}^{\circ }C$)
$\rho_{l,m}$	Density ($\mathrm{kg}\;m^{-3}$)
τ	Time (dimensionless)
$\tau_{s}$	Time of braking (dimensionless)
ζ	Spatial coordinate in axial direction (dimensionless)
$\vartheta_{l}$	Volume temperature (${}^{\circ }C$)
